# The effect of long term combined yoga practice on the basal metabolic rate of healthy adults

**DOI:** 10.1186/1472-6882-6-28

**Published:** 2006-08-31

**Authors:** MS Chaya, AV Kurpad, HR Nagendra, R Nagarathna

**Affiliations:** 1 Department of Life sciences, Swami Vivekananda Yoga Research foundation, No 19, Eknath Bhavan, Gavipuram circle, Bangalore-560019, India; 2 Institute of Population Health and Clinical Research, St John's National Academy of Health Sciences, Bangalore 560034, India

## Abstract

**Background:**

Different procedures practiced in yoga have stimulatory or inhibitory effects on the basal metabolic rate when studied acutely. In daily life however, these procedures are usually practiced in combination. The purpose of the present study was to investigate the net change in the basal metabolic rate (BMR) of individuals actively engaging in a combination of yoga practices (asana or yogic postures, meditation and pranayama or breathing exercises) for a minimum period of six months, at a residential yoga education and research center at Bangalore.

**Methods:**

The measured BMR of individuals practicing yoga through a combination of practices was compared with that of control subjects who did not practice yoga but led similar lifestyles.

**Results:**

The BMR of the yoga practitioners was significantly lower than that of the non-yoga group, and was lower by about 13 % when adjusted for body weight (P < 0.001). This difference persisted when the groups were stratified by gender; however, the difference in BMR adjusted for body weight was greater in women than men (about 8 and 18% respectively). In addition, the mean BMR of the yoga group was significantly lower than their predicted values, while the mean BMR of non-yoga group was comparable with their predicted values derived from 1985 WHO/FAO/UNU predictive equations.

**Conclusion:**

This study shows that there is a significantly reduced BMR, probably linked to reduced arousal, with the long term practice of yoga using a combination of stimulatory and inhibitory yogic practices.

## Background

Yoga is an ancient technique practiced by sages and yogis as a desirable and healthy way of life. The very meaning of yoga is to achieve a balance within the internal and external environment, thereby seeking to attain mental, spiritual and physical well-being. This is made possible through the practice of "Pranayama" or breathing exercises, "Asana" or specific postures, and Meditation [1]. It is thought that practicing yoga over a period of time leads to a decrease in respiratory rate, muscular relaxation along with calming of the mind, which might be interpreted at least partly as a decreased state of arousal [2,3]. Many acute studies have demonstrated that Transcendental Meditation, Zen Meditation, Om Meditation, and Yogic Relaxation reduce the resting oxygen consumption rate, respiratory rate, heart rate, and the spontaneous galvanic skin response [4-8]. These changes are thought to be due to decreased arousal as well as decreased mental and muscular activity. It should be noted that the findings referred to above, particularly with reference to meditation, are different from those found during sleep, to the extent that these are observed in a wakeful relaxed state, and usually in the sitting posture.

In contrast to the reduced physiological and metabolic activity observed during meditation and relaxation posture types of asana, pranayamas and other specific asanas could acutely increase the metabolic rate. An increase of 19% in oxygen consumption has been observed during the practice of one type of pranayama called the Ujjayi Pranayama [9]. Breathing through a particular nostril, while performing the Surya Anuloma Viloma (right nostril breathing), has been shown to increase oxygen consumption by 28% [7]. Other specific asanas can also increase the metabolic rate transiently over the short term [10,11]. The increase in oxygen consumption during these yogic practices is due the muscular activity associated with the posture assumed during the asana, or due to an increase in voluntary deep inhalation and exhalation during the pranayama [9].

There are at least two questions that arise from these findings. First, yoga practitioners usually combine techniques such as pranayama, asana as well as meditation in their daily yoga practice. While the acute effect of each of these techniques has been documented, the combined effect of these practices, with their stimulatory or inhibitory effect on the BMR, is unknown. Second, the experiments referred to above have documented the acute effect of yoga practices on the oxygen consumption. From a daily living viewpoint, it is desirable to demonstrate the effect of a long-term combined yoga routine on the BMR. Therefore, the primary aim of this study was to answer these questions by studying the effect of a long term (six months or greater) practice of combined techniques of yoga, on the BMR of young to middle aged men and women.

## Methods

The study was conducted at 'sVyasa', a residential yoga education and research center near Bangalore City in south India. One hundred and four subjects, 39 women and 65 men in the age range of 20–60 years, were recruited for this study after giving their informed consent. Subjects with chronic disease or those on medications or with organ system dysfunction were excluded. The subjects were divided into two groups; a yoga group (n = 55) who were those practicing combined yoga (practice of asanas, pranayamas and meditation) for the past six months or more, and a non yoga group (n = 49), who were those subjects not practicing yoga but working as volunteers or workers at sVyasa. The yoga and non yoga groups were further stratified based on gender. The number, age and physical characteristics of the subjects are provided in Table 1.

**Table 1:** Age and anthropometric characteristics of men and women belonging to yoga and non yoga groups.

**Groups**		**Yoga**	**Non Yoga**
**Total**	**N**	55	49
**Group**	**Age (y)**	34.18 ± 11.3	32.66 ± 9.77
	**Weight (kg)**	57.78 ± 8.57	59.28 ± 8.62
	**Height (M)**	1.61 ± 0.01	1.56 ± 0.18
	**BMI (kg/m^2^)**	21.61 ± 3.36	21.47 ± 2.66

**Women**	**N**	24	15
	**Age (y)**	32.9 ± 11.24	33.3 ± 9.53
	**Weight (kg)**	55.9 ± 8.2	55.3 ± 9.3
	**Height (M)**	1.58 ± 0.07	1.50 ± 0.16
	**BMI (kg/m^2^)**	22.2 ± 3.8	22.0 ± 2.8

**Men**	**N**	31	34
	**Age (y)**	35.5 ± 11.4	32.0 ± 10.0
	**Weight (kg)**	59.8 ± 9.0	63.3 ± 8.0
	**Height (M)**	1.65 ± 0.13	1.62 ± 0.21
	**BMI (kg/m^2^)**	21.02 ± 3.0	20.9 ± 2.5

Values are mean ± 1 SD.

No significant difference between groups

The yoga group practiced a mixed set of yoga techniques daily, in the form of asana (postures) and deep relaxation technique, pranayama (breathing techniques) and meditation, for at least the past 6 months or more. The asana postures started with stretching techniques followed by standing, supine, prone and sitting postures. The standing postures were the side bending triangle posture (trikonasana), forward bending (padahastasana), backward bending (ardha chakrasana) and side lateral bending (ardhakati chakrasana) techniques. The supine postures were straight leg raising and shoulder stand posture (sarvangasana), while the prone postures were locust (shalabhasana), serpent (bhujangasana) and bow (dhanurasana) postures. The sitting postures were the moon (shasankasana), hardy (vajrasana), and the half matsyendra (ardha matsyendra) postures. The asanas were followed by a deep relaxation technique, which was performed for 6 minutes with closed eyes with specific instructions relating to awareness and relaxation of different parts of the body. The pranayama phase consisted of fast breathing techniques such as forceful exhalation (kapalabhathi), and breathing through the mouth with tongue folded (shithali and shithkari), sectional breathing addressing the lower, middle and upper lobes of the lungs (vibhagiya pranayama), and a slow breathing technique or alternate nostril breathing (nadishuddhi pranayama). At the end of the pranayama, the practitioner assumed the supine posture in a totally relaxed state with closed eyes (also called the corpse posture or shavasana) for 3–6 minutes, in which the aim was to achieve an awareness of relaxation of every part of the body. Meditation practices were performed in the sitting position starting with breath awareness and relaxation. More detailed descriptions of these techniques are available in yogic texts [13].

Menstruating women performed only pranayama and meditation. The non yoga group did not practice any asana, pranayama, meditation or special techniques, but otherwise lived a life that was similar to that of the yoga group, since they were volunteers at sVyasa, were vegetarian and did not drink or smoke.

During the measurement, the subjects had an early vegetarian dinner before 1800 h the previous night, followed at least 8 hours of sleep, and reported in the fasted state to the metabolic laboratory at 0530 h the next morning. Their body weight was measured to the nearest 10 g by a digital weighing scale (Soehnle, Germany), and height was measured to the nearest 0.1 cm by using a standard stadiometer. The subjects then rested in the supine posture for 20 minutes before the measurement of BMR in a quiet, thermo neutral room. The women were assessed without regard to the stage of their menstrual cycle. The BMR was measured by indirect calorimetry (Oxycon-Pro, Jaeger, Germany), using a face mask and breath by breath analysis of oxygen consumption (VO_2_) and carbon dioxide production (VCO_2_) for a period of 20 minutes; respiratory quotient (RQ) and energy expenditure (EE) were calculated from these variables [14]. Other respiratory variables collected were the ventilation volume and the breath flow rate. The machine was calibrated daily for flow volume and gas analysis by using certified gases (5.2 % of CO_2 _and balance nitrogen, and atmospheric air, BOC, UK). The 1985 FAO/WHO/UNU prediction equations were used to obtain predicted values of BMR based on age, gender and body weight.

The data are presented as mean ± SD (standard deviation). Comparisons between the groups were performed using the Student independent t test, and analysis of covariance was used to adjust the BMR for differences in body weight between the groups. Differences were considered to be significant at p < 0.05.

## Results

The yoga group consisted of 24 women and 31 men, while the non yoga group consisted of 34 men and 15 women respectively (Table 1). There were no significant differences in age or anthropometric parameters between the groups in terms of age and body weight, even when stratified for gender. The measured and predicted BMR of the subjects is shown in Table 2. Overall, there was significant decrease (15%) in the measured BMR of the yoga group when compared with the non yoga group (p < 0.000). When stratified by gender, the measured BMR of the yoga group was lower than the measured BMR in women (16%, p < 0.001) and men (12%, p < 0.001) of the non yoga group. The significant difference in BMR persisted even after adjustment for body weight by analysis of covariance between the yoga group men and women (Table 3), and the difference between yoga and non-yoga groups was of the order of about 9 and 18% in men and women respectively. When differences were analyzed between genders within groups, women in the yoga group had a significantly lower BMR when compared to men after adjusting for body weight, but this gender difference was not observed in the non yoga group (Table 3). However, there was no significant difference between the non yoga group men and women after adjusting for body weight.

**Table 2:** BMR values in yoga and non-yoga groups with their Mean predicted values

	**Yoga n = 55**	**Non Yoga n = 49**
**BMR (Kcal/d)**	1197.6 ± 238.3 **^,+^	1420.2 ± 251.2
**BMR ^¶^**	20.6 ± 3.6 **	23.6 ± 4.6
**Predicted BMR (Kcal/d)**	1388.2 ± 169.9	1423.1 ± 151.5

Mean ± 1 SD

** P < 0.001, yoga group compared with non yoga group

^+ ^P < 0.001 BMR of subjects in each group compared with their predicted values

^¶ ^Adjusted for body weight

**Table 3:** Gender wise comparison of BMR of yoga and non yoga groups along with their predicted values.

	**Women**		**Men**	
	**Yoga (n = 24)**	**Non Yoga (n = 15)**	**Yoga (n = 31)**	**Non Yoga (n = 34)**
**BMR (Kcal/d)**	1061.0 ± 201.9^‡,^**	1275.1 ± 226.2	1303.3 ± 210.9^‡,^**	1484.3 ± 237.2
**BMR ^¶^**	19.1 ± 3.5^‡,^**	23.5 ± 5.2	21.8 ± 3.3^‡,^*	23.7 ± 4.4
**Predicted BMR (Kcal/d)**	1249.7 ± 98.5	1244.9 ± 82.1	1495.5 ± 131.5	1501.7 ± 98.7

Mean ± 1 SD

** P < 0.001, yoga group compared with non yoga group

* P < 0.05, yoga group compared with non yoga group

^‡ ^P < 0.001, BMR of subjects in each group compared with their predicted values

^¶ ^Adjusted for body weight

Table 4 shows a comparison of yoga and non yoga group stratified by gender in all the other measured respiratory parameters. In addition to the BMR difference in the yoga group described above (Table 2, 3), there was also a significant decrease in other respiratory parameters between yoga and non yoga groups, such as in VO_2_, VCO_2_, respiratory minute ventilation volume (VE, for men only) and breath flow rate (BF) (Table 4). There was no significant difference in heart rate (HR) between groups, however, there were small but significant differences in the RQ between the yoga and non yoga groups, although these differences were in different directions for men and women (Table 4).

**Table 4:** Gender wise comparisons of respiratory and cardiac parameters of yoga and non yoga group.

	**Women**		**Men**	
	**Yoga (n = 24)**	**Non Yoga (n = 15)**	**Yoga (n = 31)**	**Non Yoga (n = 34)**

**VE [l/min]**	5.5 ± 1.2	6.1 ± 1.1	6.1 ± 1.0**	7.2 ± 1.3
**BF [1/min]**	14.8 ± 3.7**	17.7 ± 2.1	12.6 ± 3.5**	15.0 ± 3.2
**VO_2 _[ml/min]**	155.4 ± 27.6**	185.4 ± 32.7	192.4 ± 28.2**	212.9 ± 33.6
**VCO_2 _[ml/min]**	142.2 ± 26.2*	160.3 ± 26.6	169.7 ± 27.7**	194.0 ± 31.7
**RQ**	0.91± 0.07*	0.87 ± 0.06	0.88 ± 0.05*	0.91 ± 0.06
**HR [beats/min]**	69.6 ± 9.0	72.1 ± 5.8	64.2 ± 9.7	64.7 ± 11.2

Mean ± SD

** P < 0.001, yoga group compared with non yoga group

* P < 0.05, yoga group compared with non yoga group

VE (minute ventilation rate), BF (breath flow), V0_2 _(volume of oxygen consumed/minute)

VCO_2 _(volume of carbon dioxide expired/minute), RQ (respiratory quotient), HR (heart rate)

When compared to predicted BMR (predicted by gender and age based equations based on body weight and height), both men and women in the yoga group showed a lower measured BMR (p < 0.001). On the other hand, the BMR of the non yoga group, taken as a whole, or stratified by gender, was comparable with their predicted values (Tables 2, 3).

## Discussions and conclusion

Yoga is a state (meaning union) which is defined as a high level of consciousness achieved through a fully rested relaxed body and a fully awake and relaxed mind [5]. The effect of yoga on body function may be related to decreased arousal or a decrease in sympathetic nervous system activity. Meditation or relaxation according to yogic scriptures is a calming of the mind, slowing of the breath and relaxation of the muscles [1-3], and this is consistent with the effect of small changes in the psychological state on heart rate, respiratory rate and energy expenditure [6,8]. Earlier studies have demonstrated that transcendental meditation, Zen meditation, Yogic meditation and certain pranayama and relaxation techniques reduce O_2 _consumption, CO_2 _elimination, metabolic rate, heart rate, pulse rate, breath rate measured immediately prior to, during and after the meditation or relaxation techniques [15-18,5,6]. Recordings of the EEG in subjects practicing transcendental meditation have also demonstrated a predominant alpha wave activity (even with eyes half open) which progressively increased in amplitude and decreased in frequency during the first stage of transcendental meditation, followed by occasional theta waves in the second stage of meditation [19]. While the voluntary cessation of the heart beat by a yogi has been recorded [20], yogic and Zen meditators could also reduce their oxygen consumption, metabolic rate, and heart rate for short periods of time by [21,16]. These acute studies suggest that the mechanism by which this may occur is a possible alteration of autonomic nervous function, and studies showing decreased arousal with yoga [22,18] support this view.

The reduction in BMR in long term yoga practitioners might be considered to be a form of adaptation through reduced arousal in healthy, well nourished men and women, which, while similar in direction, is probably different from the adaptation to chronic undernutrition [23,24]. The importance of the present study is that it sought to evaluate real life practice of yoga, which usually combines several techniques and is practiced over a period of time. It also suggests a hierarchy of effects, such that the predominant outcome with mixed yoga practice (using techniques that have a stimulatory or inhibitory effect) is one of inhibition. The difference in BMR (adjusted for body weight) was much greater for the women rather than men in this study. It is possible the yoga training was more effective in women or that women respond differently to the practice of yoga, since women can respond differently to different levels of stress [25,26]. This is not unreasonable, and certainly needs further investigation. One drawback in the present study was that the phase of the menstrual cycle of the women was not constant during the measurement period, nor was a detailed menstrual history obtained. The present study also did not measure body composition, and it is possible that the differences in BMR may disappear after correcting for body composition differences. It is difficult to ascribe a direct mechanism related to yoga through physiological means, since these were not measured. The interesting follow up to these studies is to observe how long these changes last after the cessation of yoga practice, and whether the continued practice of such a lifestyle would bring certain changes that are more persistent in nature at the cellular level. There were no large differences in RQ between the groups; although there was a small but significant difference between yoga and non yoga groups in the RQ, this was different in direction between men and women, and in general, the rounded off value of the RQ in all groups was 0.9. This is consistent with a high carbohydrate intake population, as has been shown earlier [27] and does not explain the difference between groups.

The BMR can also adapt to the nutritional status and physical activity of the individual. Metabolic adaptations in terms of a reduced BMR are known to occur in acute [24] and chronic [28,23] undernutrition. The subjects in the present study were normally nourished and did not report any acute weight change during the last six months, and the women were non-pregnant and non-lactating. Physical activity is also known to influence the BMR, for example, endurance trained athletes have been shown to have a higher BMR [29] and high intensity exercise on the previous day has been shown to increase the BMR [30], although low and moderate intensity exercises does not seem to have a similar effect [31]. Notwithstanding the latter finding, it would seem likely that yoga, which is a mild to moderate form of activity [10,11], would, if at all, increase the BMR. However, this study shows that yoga, when practiced in a combined mode, with asana, pranayama and meditation over a period of time, actually reduces the BMR. Other respiratory parameters such as minute ventilation volume and breath flow rate also were lower in the yoga group suggesting a controlled pattern of breathing. Both groups of subjects had similar resting heart rates suggesting that there was no actual change in physical fitness between the groups.

An interesting implication that underlies the reduced BMR with long term combined yoga practice is whether it creates a propensity for weight gain and fat deposition. The latter is a problem in India, in which higher adiposity is observed at a lower body mass index, and in general, urban prevalence of chronic non-communicable disease is high [32,33] In contrast, yoga is thought to be associated with positive effects on health, and this might be related to other concomitant beneficial changes in appetite, food intake and body composition. The effect of a lowered BMR on the risk for chronic disease needs to be evaluated through follow up studies, with measurements of the effect of long term combined yoga on the appetite, weight stability, sense of well-being and body composition. In conclusion, the present study demonstrates that the long term practice of yoga and meditation leads to a decline in the BMR, and this decline is seen in men and women.

## Competing interests

The author(s) declare that they have no competing interests.

## Authors' contributions

All the authors contributed equally to this article.

All authors read and approved the final manuscript.

## Pre-publication history

The pre-publication history for this paper can be accessed here:


